# Supplementary material for: Adaptive physics-informed neural operator for coarse-grained non-equilibrium flows
Source: Sci Rep. 2023 Sep 19;13:15497. doi: 10.1038/s41598-023-41039-y (PMC10509218; doi:10.1038/s41598-023-41039-y)
Supplement: Supplementary file 1 — Supplementary Information. [file 41598_2023_41039_MOESM1_ESM.pdf]

Supplementary Information to "*Adaptive physics-informed neural operator for coarse-grained non-equilibrium flows*"

Ivan Zanardi[1], Simone Venturi[1], and Marco Panesi[1,*]

[1]Center for Hypersonics and Entry Systems Studies, Department of Aerospace Engineering, University of Illinois Urbana-Champaign, Urbana, IL 61801, USA
*mpanesi@illinois.edu

ABSTRACT

This work proposes a new machine learning (ML)-based paradigm aiming to enhance the computational efficiency of non-equilibrium reacting flow simulations while ensuring compliance with the underlying physics. The framework combines dimensionality reduction and neural operators through a hierarchical and adaptive deep learning strategy to learn the solution of multi-scale coarse-grained governing equations for chemical kinetics. The proposed surrogate's architecture is structured as a tree, with leaf nodes representing separate neural operator blocks where physics is embedded in the form of multiple soft and hard constraints. The hierarchical attribute has two advantages: i) It allows the simplification of the training phase via transfer learning, starting from the slowest temporal scales; ii) It accelerates the prediction step by enabling adaptivity as the surrogate's evaluation is limited to the necessary leaf nodes based on the local degree of non-equilibrium of the gas. The model is applied to the study of chemical kinetics relevant for application to hypersonic flight, and it is tested here on pure oxygen gas mixtures. In 0-D scenarios, the proposed ML framework can adaptively predict the dynamics of almost thirty species with a maximum relative error of 4.5% for a wide range of initial conditions. Furthermore, when employed in 1-D shock simulations, the approach shows accuracy ranging from 1% to 4.5% and a speedup of one order of magnitude compared to conventional implicit schemes employed in an operator-splitting integration framework. Given the results presented in the paper, this work lays the foundation for constructing an efficient ML-based surrogate coupled with reactive Navier-Stokes solvers for accurately characterizing non-equilibrium phenomena in multi-dimensional computational fluid dynamics simulations.

S.1 Physical modeling

The gaseous mixtures considered in the proposed framework consist solely of oxygen atoms and molecules, both assumed to be in their ground electronic states. The set of pseudo-species, i, is defined as $\mathscr{I} = \left\{\mathrm{O}, \mathrm{O}_2^{(i)}\right\}$, encompassing all the possible internal energy degrees of freedom of the system.

S.1.1 Thermodynamics

The gas pressure follows from Dalton's law,

$$p = \sum_{i \in \mathscr{I}} n_i k_\mathrm{B} T \quad , \tag{S1}$$

where n_i stands for the number density of the pseudo-species i, whereas k_B denotes Boltzmann's constant. The gas density reads $\rho = \sum_{i \in \mathscr{I}} \rho_i$, where the partial densities are related to the number densities via $\rho_i = m_i n_i$, with m_i being the (particle) mass of i. The energy per unit-mass of the individual pseudo-species may be written as

$$e_i = e_{i,\mathrm{tr}} + e_{i,\mathrm{int}} + \Delta h_{i,\mathrm{f}} \quad , \tag{S2}$$

where translational contribution follows from the principle of equipartition of energy[1]:

$$e_{i,\mathrm{tr}} = \frac{3}{2} \frac{k_\mathrm{B}}{m_i} T \quad . \tag{S3}$$

The symbol $\Delta h_{i,\mathrm{f}}$ in equation (S2) denotes the formation enthalpy at 0 K, whereas the remaining term, $e_{i,\mathrm{int}}$, accounts for the internal energy degree of freedom of the pseudo-species i:

i. If the state-to-state (StS) modeling is used, the term $e_{i,\text{int}}$ represents a particular rovibrational energy state, denoted as ε_i, where $i = (s, v, J)$ with s representing the species, v representing the vibrational quantum number, and J representing the rotational quantum number.

ii. If coarse-grained modeling is used, ρ_i indicates the density of a group of states and $e_{i,\text{int}}$ can be expressed as follows:

$$e_{i,\text{int}} = e_P = \sum_{i \in \mathscr{I}_P} \frac{q_i(T_P)}{Q_P(T_P)} \varepsilon_i \quad , \tag{S4}$$

with the additional new terms appearing in equation (S4) described in Section S.1.2.

iii. If multi-temperature (MT) models[2] are employed, $e_{i,\text{int}}$ accounts for the energy of *thermalized* internal degrees of freedom (*e.g.*, rotation, vibration). For a conventional two-temperature (2T) formulation[2,3], which is a particular class of MT models, the expression of $e_{i,\text{int}}$ for a diatomic molecule described by the rigid-rotor and harmonic oscillator models is[4,5]:

$$e_{i,\text{int}} = e_{i,\text{r}}(T_\text{r}) + e_{i,\text{v}}(T_\text{v}) \quad , \tag{S5}$$

with

$$e_{i,\text{r}}(T_\text{r}) = \frac{k_\text{B}}{m_i} T_\text{r} \quad , \tag{S6}$$

$$e_{i,\text{v}}(T_\text{v}) = \frac{k_\text{B}}{m_s} \frac{\theta_s^\text{v}}{\exp(\theta_s^\text{v}/T_\text{v}) - 1} \quad , \tag{S7}$$

where T_r and T_v are, respectively, the rotational and vibrational temperatures, whereas θ_s^v is the characteristic vibrational temperature. In Park's two-temperature model, the fast equilibration between rotational and translational energy mode is assumed (*i.e.*, $T_\text{r} = T$).

Collecting the above formulae, the energy per unit-mass of the gas as a whole can be defined as follows:

$$e = \sum_{i \in \mathscr{I}} Y_i \left(e_{i,\text{tr}} + e_{i,\text{int}} + \Delta h_{i,\text{f}}\right) \quad , \tag{S8}$$

where the mass fractions are $Y_i = \rho_i/\rho$.

S.1.2 Coarse-grained modeling

This work employs a log-linear form of the distribution function to represent the population within each individual bin, which results in a thermalized local Boltzmann distribution defined as follows:

$$\mathscr{F}_P^i(\varepsilon_i): \quad \log\left(\frac{g_i}{n_i}\right) = \alpha_P + \beta_P \varepsilon_i \quad . \tag{S9}$$

The bin-specific coefficients α_P and β_P are formulated in terms of the macroscopic constraints, total bin population n_P and energy e_P,

$$n_P = \sum_{i \in \mathscr{I}_P} n_i \ , \quad e_P = \sum_{i \in \mathscr{I}_P} n_i \varepsilon_i \quad , \tag{S10}$$

where $\mathscr{I}_P$ indicates the set of rovibrational states contained in the P-th group. The bin internal temperature T_P can be used instead of β_P to characterize the bin distribution function,

$$\beta_P = \frac{1}{k_B T_P} \quad , \tag{S11}$$

while the coefficient α_P can then be defined as follows:

$$\alpha_P = \log\left(\frac{Q_P}{n_P}\right) \quad , \tag{S12}$$

where Q_P is the group internal partition function,

$$Q_P(T_P) = \sum_{i \in \mathscr{I}_P} q_i(T_P) \quad , \tag{S13}$$

with

$$q_i(T_P) = g^\text{e} g_i \exp\left(-\frac{\varepsilon_i}{k_B T_P}\right) \tag{S14}$$

being the i-th level contribution, k_B the Boltzmann's constant, and g^e the degeneracy of the electronic ground state.

S.1.2.1 Zero-dimensional macroscopic equations

Considering O_2+O system, as the group temperatures T_P are assumed to be equal to the translational temperature T, only the zeroth-order moment of the StS master equations[6,7] is required to model the reactor dynamics:

$$\begin{cases} \dfrac{dn_P}{dt} = \Omega_P^0 = -\displaystyle\sum_{\mathscr{I}_Q \in \mathscr{I}_{O_2}} K_{PQ}^E n_P n_O + \sum_{\mathscr{I}_Q \in \mathscr{I}_{O_2}} K_{QP}^E n_Q n_O - K_P^D n_P n_O + K_P^R n_O^3 \qquad \forall\ \mathscr{I}_P \in \mathscr{I}_{O_2} \\ \dfrac{dn_O}{dt} = \Omega_O^0 = \displaystyle\sum_{\mathscr{I}_P \in \mathscr{I}_{O_2}} K_P^D n_P n_O - \sum_{\mathscr{I}_P \in \mathscr{I}_{O_2}} K_P^R n_O^3 \end{cases} , \tag{S15}$$

with $\mathscr{I}_{O_2}$ being the set of pseudo-species, *i.e.*, groups, of O_2. The group-specific rate coefficients, K_{PQ}^E and K_P^D, are obtained from the state-specific ones, k_{ij}^E and k_i^D, as a weighted average based on the Boltzmann distribution function over $\mathscr{I}_P$:

$$K_{PQ}^E(T,T_P) = \sum_{i \in \mathscr{I}_P} \sum_{j \in \mathscr{I}_Q} \frac{q_i(T_P)}{Q_P(T_P)} k_{ij}^E(T) \quad , \tag{S16}$$

$$K_P^D(T,T_P) = \sum_{i \in \mathscr{I}_P} \frac{q_i(T_P)}{Q_P(T_P)} k_i^D(T) \quad . \tag{S17}$$

S.2 Neural operators

S.2.1 DeepONet

S.2.1.1 Vanilla architecture

The vanilla version of the DeepONet consists of one branch net and one trunk net. To account for the problem's multi-dimensionality, the feature embedding $\boldsymbol{\alpha}$ (and equivalently $\boldsymbol{\phi}$) has a dimension of $p \times D$, where p is the number of modes (in a POD sense) and D is the number of output variables. To ensure a continuous and differentiable representation of the output functions, the branch and trunk network outputs are split into D p-dimensional vectors, which are merged together via dot product as follows:

$$\widehat{G}^{(i)}(\boldsymbol{u})(\boldsymbol{y}) = \sum_{k=(i-1)p+1}^{ip} \alpha_k(\boldsymbol{u})\phi_k(\boldsymbol{y}) \qquad \text{for} \;\; i = 1,\ldots,D \;\; . \tag{S18}$$

To ensure a fair comparison, the vanilla DeepONet, summarized in table S1, has been designed to have almost the same number of parameters (231 388) as the CG-DeepONets described in Section S.2.2, and it has been trained and tested on the same datasets. The entirely optimization has been performed under identical conditions in terms of hyper-parameters, including number of epochs, optimizer type, learning rate, and regularization. Table S2 presents the four largest errors of the inferred

Sub-networks	Type	Layers Width	σ
Branch	FNN	[240, 240, 224]	tanh×2 + linear
Trunk	FNN	[240, 240, 224]	tanh×2 + linear

Table S1. *Vanilla DeepONet architecture.* FNN is the conventional feed-forward neural network, and σ are the activation functions.

solution using the vanilla DeepONet, following the same procedure described in the Manuscript Section "*Accuracy*". In the table, the apex refers to the O_2 group. The results demonstrate that although the errors are within acceptable limits, they are nearly double compared to the ones reported in Table 3 in the manuscript, indicating that the vanilla DeepONet does not perform as well as the CG-DeepONets on this particular problem. Furthermore, the vanilla DeepONet cannot perform adaptive inference, which is essential for achieving increased speedup in the prediction phase.

S.2.1.2 Shared-trunk architecture

As depicted in Figure S1, the shared-trunk architecture is characterized by multiple branches, each corresponding to an output variable and a shared trunk network. This design allows for computational efficiency, as the shared trunk can be used for multiple output variables. However, it is effective only when the dynamics of the modeled variables are similar enough to share the same basis, as discussed in reference [8].

Group	Rel. error [%]
$\widehat{Y}^{(27)}$	6.80 ± 4.61
$\widehat{Y}^{(26)}$	6.55 ± 4.39
$\widehat{Y}^{(24)}$	6.43 ± 4.12
$\widehat{Y}^{(23)}$	6.42 ± 3.88

Table S2. *Vanilla DeepONet test error.* The four highest mean relative L^2-norm testing errors (with standard deviations) of the trained vanilla DeepONet.

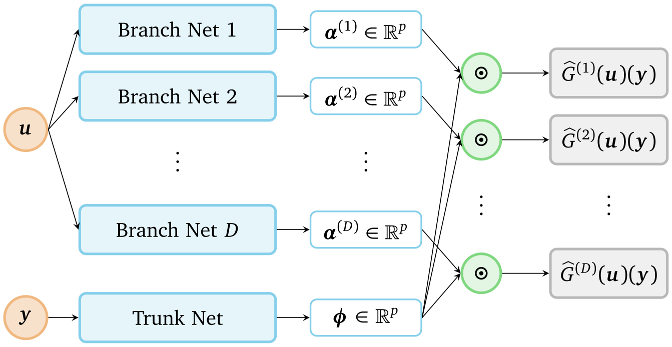

Figure S1. *Multi-output DeepONet.* The modified architecture consists of multiple "branch nets" (one for each output) for extracting latent representations of the input functions and one "trunk net" for extracting latent representations of the input coordinates at which the output functions are evaluated.

S.2.2 Multi-scale hierarchical coarse-grained model

S.2.2.1 Hyper-parameter settings

Table S3 summarizes the CG-DeepONets architecture, where a modified version of the DeepONet proposed by Wang *et al.* [Eqs. (3.23)-(3.29) in reference [9]] is used. The network is trained via mini-batch stochastic gradient descent for 10^4 iterations using the Adam optimizer for each step (a-d) described in the Manuscript Section "*Training Strategy*". The last step using physics-informed optimization techniques has been performed for 5×10^3 epochs. For each training step, to obtain a set of training and validation data, $2 \times N$ initial conditions have been sampled using the Latin Hypercube strategy with $N = 2048$. Half of them have been selected as training scenarios using the stratified sampling method, and the remaining half as validation. For each i-th initial condition, $P = 128$ and $P = 32$ data points for training and validation have been log-uniformly sampled in time. To generate the test data set, we randomly sampled 100 unseen initial conditions and obtained the corresponding numerical solutions by integrating the ODE using a conventional numerical integrator.

It has to be mentioned that an input transformation layer is used to modify the input features. For the trunk net, the time t has been linearly scaled by a factor of 10^7, while for the branch net, the temperature T has been normalized between 0 and 1. The total number of parameters of the network is 230 106.

S.2.2.2 Extra test cases

Figure S2 shows extra testing cases, similarly to what has been shown in the Manuscript Section "*Inference*".

S.2.2.3 Loss histories

In this section, only the last and most interesting hybrid training step of the strategy described in the Manuscript Section "*Training Strategy*" is presented. Figures S3(a-b) show the training and validation losses, respectively, while Figure S3(c) shows the evolution of the weights coefficients λ_i, automatically tuned every 50 iterations with the learning rate annealing technique described by Alg. 2.1 in reference [10]. λ_r is fixed and equal to 1 since the ODE residual loss, $\mathscr{L}_r$, is used as the reference value for computing λ_d and λ_{ic}, while the hyper-parameter α of the tuning procedure has been set to 0.7.

	# DeepONets	Single DeepONet				
		Sub-networks	Type	p	Layers Width	σ
Timescale 1	1	2 Branches<br>1 Trunk	ResFNN<br>ResFNN	8	[32, 32, p]<br>[32, 32, p]	tanh×3<br>tanh×3
Timescale 2	1	3 Branches<br>1 Trunk	ResFNN<br>ResFNN	16	[48, 48, p]<br>[48, 48, p]	tanh×3<br>tanh×3
Timescale 3	3	3 Branches<br>1 Trunk	ResFNN<br>ResFNN	16	[48, 48, p]<br>[48, 48, p]	tanh×3<br>tanh×3
Timescale 4	9	3 Branches<br>1 Trunk	ResFNN<br>ResFNN	16	[48, 48, p]<br>[48, 48, p]	tanh×3<br>tanh×3

Table S3. *CG-DeepONets architecture.* p is the dimension of the features embedding, ResFNN is a novel neural network architecture proposed by Wang *et al.* [Eqs. (2.33)-(2.37) in reference [10]], and σ are the activation functions.

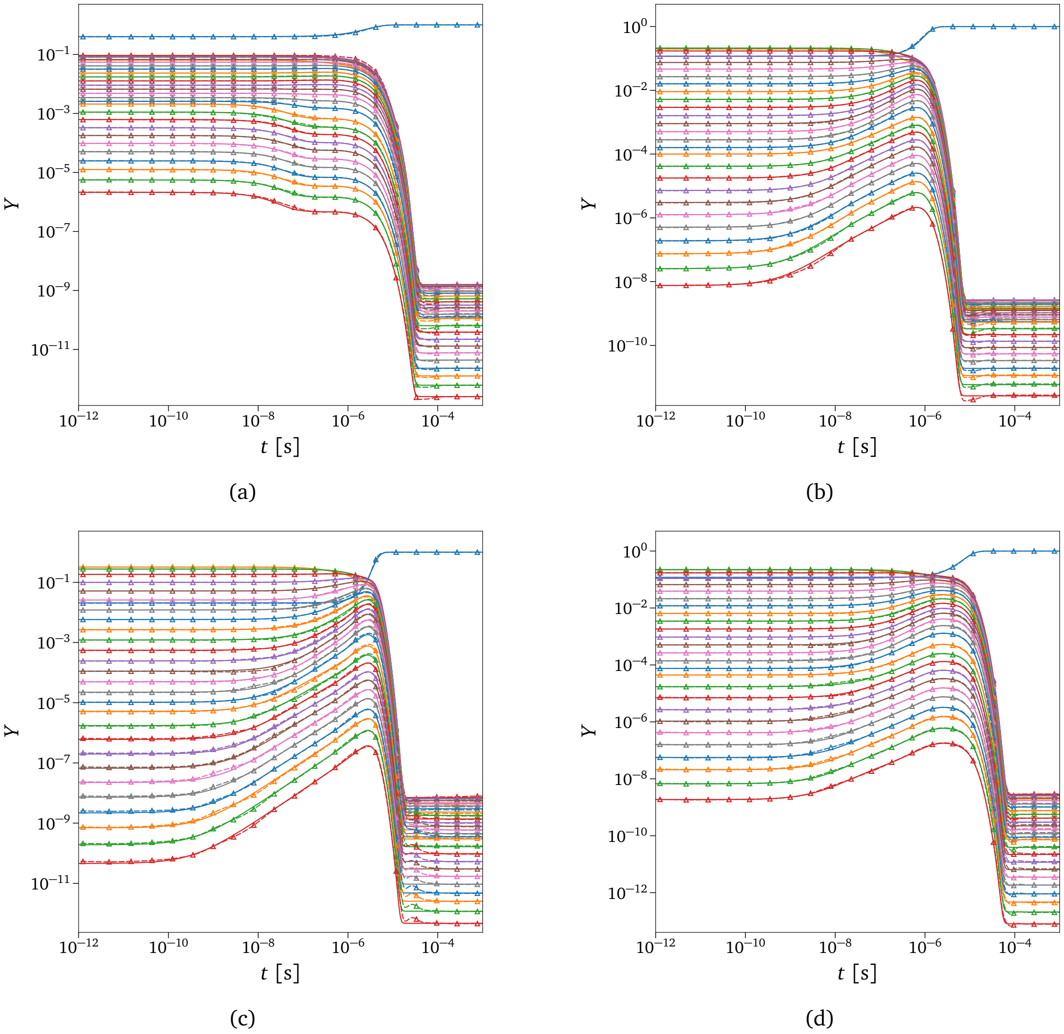

Figure S2. *Extra test cases for CG-DeepONets.*

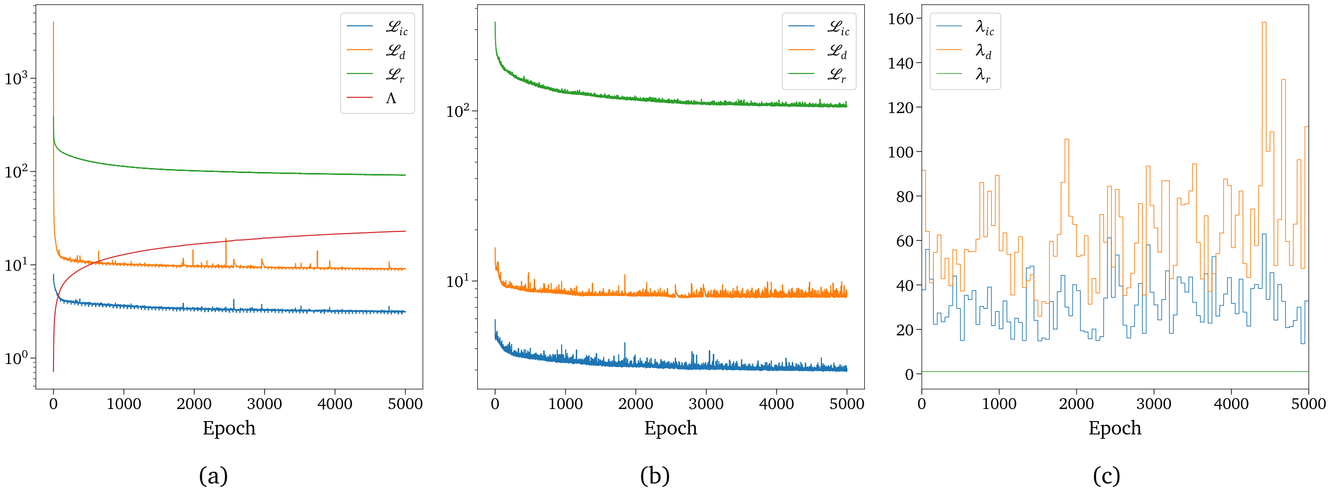

Figure S3. *Loss histories of the last physics-informed training step.* (a) Training losses. (b) Validation losses. (c) Loss weighting coefficients.

S.2.3 Adaptive inference

S.2.3.1 Hyper-parameter settings

Table S4 summarizes the Neq-DeepONets architecture. We trained the model by employing a similar strategy used for the CG-DeepONets, except that all the timescales have been trained simultaneously in this case. The version of the DeepONet used to construct the controller-acting surrogate is an augmented version called *flexDeepONet* proposed by Venturi and Casey [figure (8) in reference [8]]. The network is trained via mini-batch stochastic gradient descent for 2×10^4 iterations using the Adam optimizer and the mean absolute percentage error as the loss function. How concerns the input transformation layer,

	# DeepONets	Single DeepONet				
		Sub-networks	Type	p	Layers Width	σ
Timescale 1	1	1 Branch	FNN	8	[32, 32, p]	tanh×2 + linear
		1 Trunk	FNN		[32, 32, p]	tanh×2 + linear
		1 PreNet	FNN	-	[16, 16, 2]	tanh×2 + linear
Timescale 2	1	3 Branches	FNN	16	[48, 48, p]	tanh×2 + linear
		1 Trunk	FNN		[48, 48, p]	tanh×2 + linear
		1 PreNet	FNN	-	[16, 16, 2]	tanh×2 + linear
Timescale 3	3	3 Branches	FNN	16	[48, 48, p]	tanh×2 + linear
		1 Trunk	FNN		[48, 48, p]	tanh×2 + linear
		1 PreNet	FNN	-	[16, 16, 2]	tanh×2 + linear

Table S4. *Neq-DeepONets architecture.* p is the dimension of the features embedding, FNN is the conventional feed-forward neural network, and σ are the activation functions.

for the trunk net, the time t has been linearly scaled by a factor of 10^7 and then log-transformed, while for the branch net, the temperature T has been normalized between 0 and 1. An exponential transformation function is applied to the output of each DeepONet. The total number of parameters of the network is 75 487.

S.2.3.2 Inference solution and accuracy

In figure S5, a comparison between the exact and inferred solutions of the trained model is presented for different test cases, while table S5 reports the four highest errors of the inferred solution, similar to what has been done in Section the Manuscript Section "*Accuracy*".

S.2.3.3 Adaptive inference algorithm

Algorithm S1 presents in detail all the steps of the adaptive technique used to get the inferred solutions.

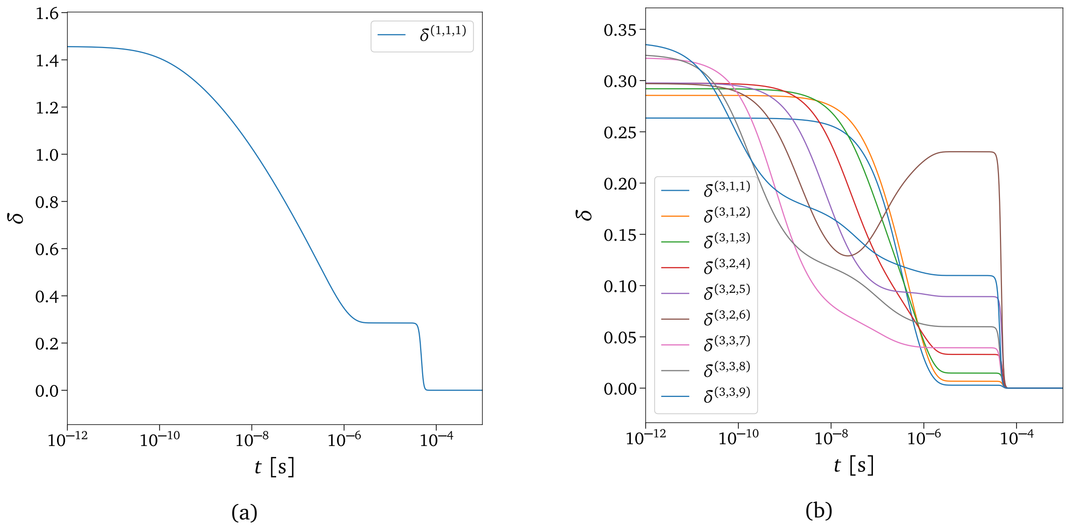

Figure S4. *Underpredicted non-equilibrium metrics.* Dynamics of underpredicted non-equilibrium metrics evaluated by reconstructing the 27 groups with predictions from *Timescale 1* (a) and *Timescale 3* (b) for the same test case shown in figure 9.

Neq. metric	Rel. error [%]
$\delta^{(3,2,6)}$	1.81 ± 1.50
$\delta^{(3,1,3)}$	1.48 ± 1.34
$\delta^{(2,1,2)}$	1.46 ± 1.25
$\delta^{(3,2,5)}$	1.44 ± 1.24

Table S5. *Neq-DeepONets test error.* The four highest mean relative L^2-norm testing errors (with standard deviations) of the trained Neq-DeepONets surrogate.

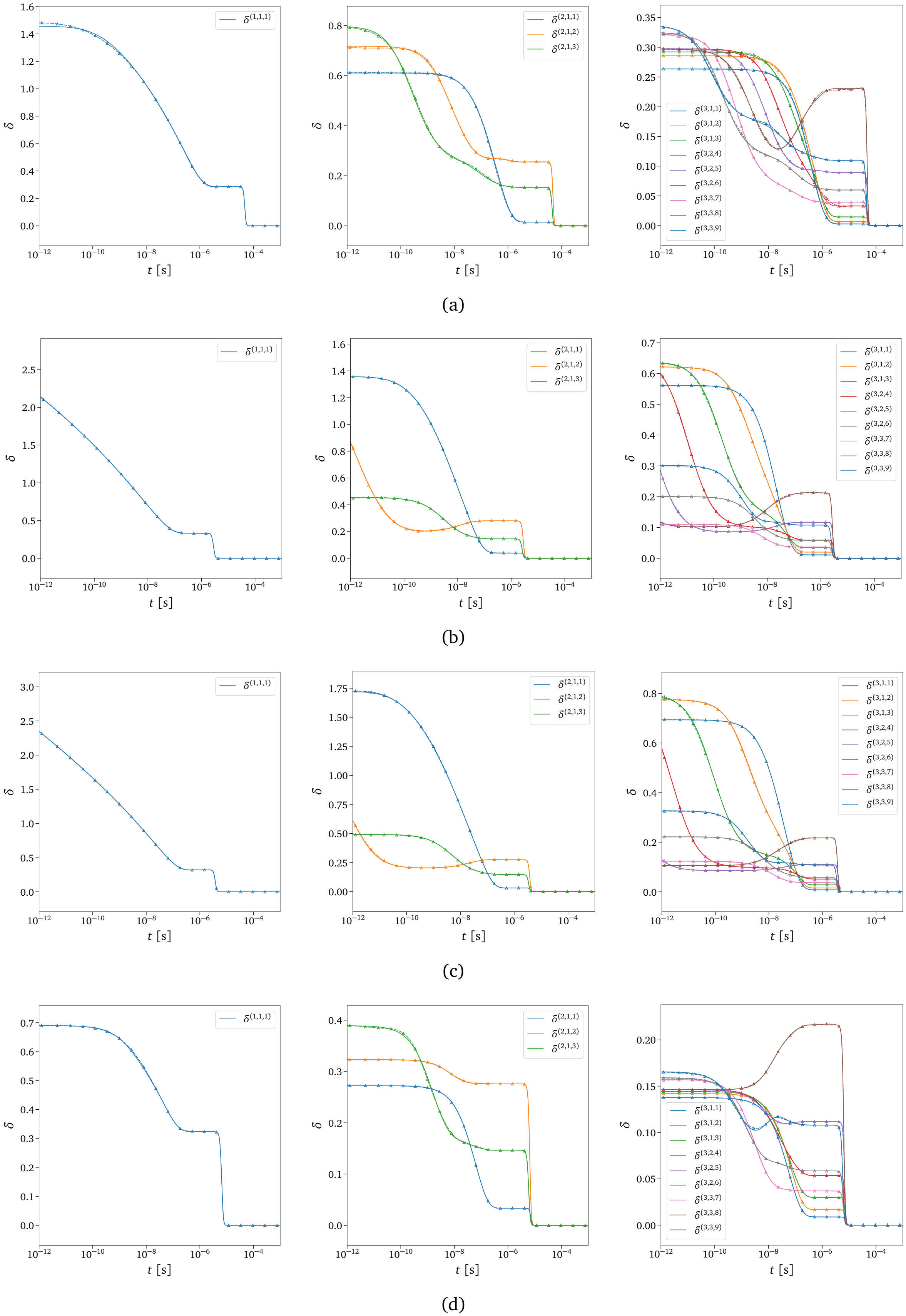

Figure S5. *Test cases for Neq-DeepONets.* Case (a) is the one shown in figure 9.

Algorithm S1: Adaptive inference

Input: Initial conditions matrix and times instants vector

$$X = \left[\ \left\{ \left(T^i, \rho^i, \boldsymbol{Y}^i_{\mathrm{O}_{20}} \right) \right\}_{i=1}^{n} \in \mathbb{R}^{n\times(2+\mathscr{N_G})}, \ \left\{ t^i \right\}_{i=1}^{n} \in \mathbb{R}^n \ \right]$$

Output: Mass fractions matrix

$$Y = \left\{ \widehat{\boldsymbol{Y}}^i \right\}_{i=1}^{n} \in \mathbb{R}^{n\times(1+\mathscr{N_G})}$$

define TS = Number of timescales considered
define δ_{tol} = Underpredicted non-equilibrium tolerance metric

Step 1: Evaluate underpredicted non-equilibrium metric
for $ts = 1,\dots,TS-1$ **do**
  define $\mathscr{N_G}_{ts}$ = Number of groups in *Timescale ts*
  if $ts = 1$ **then**
    Compute $\boldsymbol{\delta}^{(1,1,1)} \in \mathbb{R}^n$ with Neq-DeepONet$^{(1,1)}$
  else
    if $ts \neq TS-1$ **and** $all\left(\left\{\delta_i^{(ts-1,\cdot,P)} \leq \delta_{tol}\right\}_{i=1}^{n}\right) \ \forall\ P = 1,\dots,\mathscr{N_G}_{ts-1}$ **then**
      break
    else
      for $P = 1,\dots,\mathscr{N_G}_{ts-1}$ **do**
        define $\mathscr{N}_p$ = Number of micro-groups p contained in macro-group P, $\mathscr{I}_p \subset \mathscr{I}_P$
        if $any\left(\left\{\delta_i^{(ts-1,\cdot,P)} > \delta_{tol}\right\}_{i=1}^{n}\right)$ **then**
- Mask out input data points for which $\left\{\delta_i^{(ts-1,\cdot,P)} \leq \delta_{tol}\right\}_{i=1}^{m}$ with $m < n$
- Compute $\boldsymbol{\delta}^{(ts,\cdot,p)} \in \mathbb{R}^{n-m} \ \forall\ \mathscr{I}_p \subset \mathscr{I}_P$ with Neq-DeepONet$^{(ts,P)}$
- Assign $\boldsymbol{\delta}^{(ts,\cdot,p)} = \boldsymbol{0} \in \mathbb{R}^m \ \forall\ \mathscr{I}_p \subset \mathscr{I}_P$

        else
          Assign $\boldsymbol{\delta}^{(ts,\cdot,p)} = \boldsymbol{0} \in \mathbb{R}^n \ \forall\ \mathscr{I}_p \subset \mathscr{I}_P$

Step 2: Employ computed underpredicted non-equilibrium metric to evaluate mass fractions
for $ts = 1,\dots,TS$ **do**
  define $\mathscr{N_G}_{ts}$ = Number of groups in *Timescale ts*
  if $ts = 1$ **then**
    Compute $\left\{\widehat{\boldsymbol{Y}}_{\mathrm{O}}, \widehat{\boldsymbol{Y}}_{\mathrm{O}_2}^{(1,1,1)}\right\} \in \mathbb{R}^{n\times 2}$ with CG-DeepONet$^{(1,1)}$
  else
    if $ts \neq TS$ **and** $all\left(\left\{\delta_i^{(ts-1,\cdot,P)} \leq \delta_{tol}\right\}_{i=1}^{n}\right) \ \forall\ P = 1,\dots,\mathscr{N_G}_{ts-1}$ **then**
      Reconstruct $\widehat{\boldsymbol{Y}}_{\mathrm{O}_2}^{(TS,\cdot,p)} \in \mathbb{R}^n \ \forall\ \mathscr{I}_p \subset \mathscr{I}_P$ from $\widehat{\boldsymbol{Y}}_{\mathrm{O}_2}^{(ts-1,\cdot,P)} \in \mathbb{R}^n \ \forall\ P = 1,\dots,\mathscr{N_G}_{ts-1}$ by employing the Boltzmann distribution function
      break
    else
      for $P = 1,\dots,\mathscr{N_G}_{ts-1}$ **do**
        define $\mathscr{N}_p$ = Number of micro-groups p contained in macro-group P, $\mathscr{I}_p \subset \mathscr{I}_P$
        if $any\left(\left\{\delta_i^{(ts-1,\cdot,P)} > \delta_{tol}\right\}_{i=1}^{n}\right)$ **then**
- Mask out input data points for which $\left\{\delta_i^{(ts-1,\cdot,P)} \leq \delta_{tol}\right\}_{i=1}^{m}$ with $m < n$
- Compute $\widehat{\boldsymbol{Y}}_{\mathrm{O}_2}^{(ts,\cdot,p)} \in \mathbb{R}^{n-m} \ \forall\ \mathscr{I}_p \subset \mathscr{I}_P$ with CG-DeepONet$^{(ts,P)}$
- Reconstruct $\widehat{\boldsymbol{Y}}_{\mathrm{O}_2}^{(ts,\cdot,p)} \in \mathbb{R}^m \ \forall\ \mathscr{I}_p \subset \mathscr{I}_P$ from $\widehat{\boldsymbol{Y}}_{\mathrm{O}_2}^{(ts-1,\cdot,P)} \in \mathbb{R}^m$ by employing the Boltzmann distribution function

        else
          Reconstruct $\widehat{\boldsymbol{Y}}_{\mathrm{O}_2}^{(ts,\cdot,p)} \in \mathbb{R}^n \ \forall\ \mathscr{I}_p \subset \mathscr{I}_P$ from $\widehat{\boldsymbol{Y}}_{\mathrm{O}_2}^{(ts-1,\cdot,P)} \in \mathbb{R}^n$ by employing the Boltzmann distribution function

S.3 One-dimensional numerical experiment

In this section, the construction of the surrogates used in the one-dimensional numerical experiment is described, which involves the following steps:

i. Running the exact solution using the computational framework described in Section S.3.2 and the configuration described in the Manuscript Section "*One-dimensional numerical experiment*".

ii. Collecting all the possible thermochemical states experienced by the gas in the 1-D simulation and fitting a 29-dimensional multivariate Gaussian-based kernel density estimator (KDE) to the data, which includes temperature, T, and densities ρ_i of O and the 27 groups of O_2.

iii. Sampling $N = 5120$ initial thermochemical states from the constructed KDE for training and validation, and using $N = 100$ states for testing. Then, performing 0-D simulations for all the sampled initial states.

iv. Conducting a singular value decomposition (SVD) analysis on the trajectories obtained from the previous step to estimate the number of modes required for modeling each timescale in the CG-DeepONets surrogate[8]. Similarly, utilizing Equation (21) to obtain data for Neq-DeepONets surrogate from the generated trajectories, and performing the same SVD analysis.

v. Constructing the datasets for CG-DeepONets and Neq-DeepONets by sampling 72 points for training and 18 points for validation from the previously generated trajectories in a time window of $[10^{-10}, 10^{-6}]$ s, which encompasses the time steps used in the numerical experiment.

vi. Training both models, CG-DeepONets and Neq-DeepONets, following the procedures described in Section S.2.2.1 and Section S.2.3.1, respectively. However, in this case, the hybrid step described in the Manuscript Section "*Training Strategy*" is not performed.

S.3.1 Surrogate hyper-parameter settings

The architectures of CG-DeepONets and Neq-DeepONets employed for the 1-D test case are summarized in Table S6 and Table S7, respectively. Each trunk of the Neq-DeepONets has been fitted with a radial basis function (RBF) interpolator after training to accelerate the network evaluation. The version of DeepONet used to construct the CG-DeepONets surrogate is the *flexDeepONet* proposed by Venturi and Casey[8]. In this case, a unique global *PreNet* for each modeled *Timescale* is used, constructed with a feedforward neural network (FNN) architecture consisting of layers with widths [16, 16, 2] and activation functions [*tanh, tanh, linear*]. Both initial conditions and time inputs are log-transformed in both surrogates. Additionally, an exponential transformation function is applied to the output of each DeepONet in the Neq-DeepONets surrogate, as well as to the one modeling the temperature in the CG-DeepONets surrogate.

S.3.2 Computational framework

To perform the numerical experiments presented in this work, three different software are used:

i. HEGEL (High-fidElity tool for maGnEto-gasdynamics simuLations), a parallel multi-block structured fluid solver for LTE/NLTE plasmas written in modern object oriented `Fortran` 2008[11–13].

ii. PLATO (PLAsmas in Thermodynamic nOn-equilibrium), a physico-chemical library to evaluate thermodynamic, transport and optical properties as well as source terms due to NLTE collisional and radiative processes[11–13].

iii. PyCOMET (Physics-informed machine learning for scientific computing and operator discovery) is a TensorFlow-based[14] machine learning library that is used to construct neural operators and generic deep neural network (DNN)-based surrogates for scientific computing[15,16]. Previous approaches to integrating machine learning models into computational fluid dynamics (CFD) codes have included remote function calls from legacy `Fortran` codes to modern machine learning libraries, re-implementation of the full fluid solver in TensorFlow, or direct embedding of the network into the code. In this work, the approach used leverages the in-house `Fortran` and `C++` PyCOMET interfaces, which rely on CppFlow[17], a `C++` wrapper of the TensorFlow `C` API. One significant benefit of this approach is its flexibility, as the interfaces can read and import any network or generic ML-based architectures into external codes without requiring complicated supplementary coding, and support both CPU and GPU operations.

The PLATO library is responsible for performing the integration of the reactive step in equation (9) employed for evaluating the speedup in the one-dimensional shock case scenario (see the Manuscript Section "*One-dimensional shock case scenario*"). The ODE integrator employed is the second-order backward differentiation formula (BDF-2) from the LSODE (Livermore Solver for Ordinary Differential Equations) library[18], with an absolute tolerance and a relative tolerance set to 10^{-9} and 10^{-6}, respectively.

	# PreNets	# DeepONets	Single DeepONet				
			Sub-networks	Type	p	Layers Width	σ
Temperature	1	1	1 Branches<br>1 Trunk	FNN	4	[6p, 6p, p]	tanh×2 + linear
Timescale 1	1	1	2 Branches<br>1 Trunk	FNN	4	[4p, 4p, p]	tanh×2 + linear
Timescale 2	1	1	3 Branches<br>1 Trunk	FNN	12	[4p, 4p, p]	tanh×2 + linear
Timescale 3	1	1	3 Branches<br>1 Trunk	FNN	4	[4p, 4p, p]	tanh×2 + linear
		1	3 Branches<br>1 Trunk		8		
		1	3 Branches<br>1 Trunk		12		
Timescale 4	1	3	3 Branches<br>1 Trunk	FNN	4	[4p, 4p, p]	tanh×2 + linear
		3	3 Branches<br>1 Trunk		8		
		3	3 Branches<br>1 Trunk		12		

Table S6. *CG-DeepONets architecture for 1-*D *shock case scenario.* p is the dimension of the features embedding, FNN is the conventional feed-forward neural network, and σ are the activation functions. Total parameters: 125 460.

	# DeepONets	Single DeepONet				
		Sub-networks	Type	p	Layers Width	σ
Timescale 1	1	1 Branches<br>1 Trunk	FNN	8	[24, 24, p]	tanh×2 + linear
Timescale 2	1	3 Branches<br>1 Trunk	FNN	16	[36, 36, p]	tanh×2 + linear
Timescale 3	3	3 Branches<br>1 Trunk	FNN	8	[24, 24, p]	tanh×2 + linear

Table S7. *Neq-DeepONets architecture for 1-*D *shock case scenario.* p is the dimension of the features embedding, FNN is the conventional feed-forward neural network, and σ are the activation functions. Total parameters: 30 325.

References

1. Callen, H. B. & Scott, H. L. Thermodynamics and an Introduction to Thermostatistics, 2nd ed. *Am. J. Phys.* **66**, 164–167, DOI: 10.1119/1.19071 (1998).
2. Park, C. *Nonequilibrium Hypersonic Aerothermodynamics* (Wiley, New York, 1990).
3. Park, C. Review of chemical-kinetic problems of future NASA missions. I - Earth entries. *J. Thermophys. Heat Transf.* **7**, 385–398, DOI: 10.2514/3.431 (1993).
4. Vincenti, W. G. & Kruger, C. H. *Introduction to physical gas dynamics*, vol. 1 (Wiley, New York, 1965).
5. Anderson, J. D. *Hypersonic and High-Temperature Gas Dynamics, Third Edition* (American Institute of Aeronautics and Astronautics, Inc., Reston, VA, 2019).
6. Panesi, M., Jaffe, R. L., Schwenke, D. W. & Magin, T. E. Rovibrational internal energy transfer and dissociation of $N_2(^1\Sigma_g^+)$-$N(^4S_u)$ system in hypersonic flows. *The J. Chem. Phys.* **138**, 044312, DOI: 10.1063/1.4774412 (2013).
7. Panesi, M., Munafò, A., Magin, T. E. & Jaffe, R. L. Nonequilibrium shock-heated nitrogen flows using a rovibrational state-to-state method. *Phys. Rev. E* **90**, 013009, DOI: 10.1103/PhysRevE.90.013009 (2014).
8. Venturi, S. & Casey, T. SVD perspectives for augmenting DeepONet flexibility and interpretability. *Comput. Methods Appl. Mech. Eng.* **403**, 115718, DOI: 10.1016/j.cma.2022.115718 (2023).
9. Wang, S., Wang, H. & Perdikaris, P. Improved Architectures and Training Algorithms for Deep Operator Networks. *J. Sci. Comput.* **92**, 35, DOI: 10.1007/s10915-022-01881-0 (2022).
10. Wang, S., Teng, Y. & Perdikaris, P. Understanding and mitigating gradient flow pathologies in physics-informed neural networks (2020). 2001.04536.
11. Munafò, A., Alberti, A., Pantano, C., Freund, J. B. & Panesi, M. A computational model for nanosecond pulse laser-plasma interactions. *J. Comput. Phys.* **406**, 109190, DOI: 10.1016/j.jcp.2019.109190 (2020).
12. Alberti, A., Munafò, A., Pantano, C. & Panesi, M. Self-Consistent Computational Fluid Dynamics of Supersonic Drag Reduction via Upstream-Focused Laser-Energy Deposition. *AIAA J.* **59**, 1214–1224, DOI: 10.2514/1.J059612 (2021).
13. Alberti, A. *et al.* Non-equilibrium plasma generation via nano-second multi-mode laser pulses. *J. Appl. Phys.* **131**, 033102, DOI: 10.1063/5.0065999 (2022).
14. Abadi, M. *et al.* TensorFlow: Large-Scale Machine Learning on Heterogeneous Systems, DOI: 10.48550/ARXIV.1603.04467 (2016).
15. Zanardi, I., Venturi, S. & Panesi, M. Towards Efficient Simulations of Non-Equilibrium Chemistry in Hypersonic Flows: A Physics-Informed Neural Network Framework. In *AIAA SCITECH 2022 Forum*, DOI: 10.2514/6.2022-1639 (American Institute of Aeronautics and Astronautics, Reston, Virginia, 2022).
16. Sharma Priyadarshini, M., Venturi, S., Zanardi, I. & Panesi, M. Efficient Quasi-Classical Trajectory Calculations by means of Neural Operator Architectures, DOI: 10.26434/chemrxiv-2022-fs3rv (2022).
17. Izquierdo, S. cppflow: Run TensorFlow models in C++ without installation and without Bazel, DOI: 10.5281/zenodo.7107618 (2019).
18. Radhakrishnan, K. & Hindmarsh, A. C. Description and use of LSODE, the Livemore Solver for Ordinary Differential Equations. Tech. Rep., Lawrence Livermore National Laboratory (LLNL), Livermore, CA (1993). DOI: 10.2172/15013302.
